# Social Environment Shapes the Speed of Cooperation

**DOI:** 10.1038/srep29622

**Published:** 2016-07-20

**Authors:** Akihiro Nishi, Nicholas A. Christakis, Anthony M. Evans, A. James O’Malley, David G. Rand

**Affiliations:** 1 Department of Epidemiology, UCLA Fielding School of Public Health, Los Angeles, CA 90095 USA; 2 Yale Institute for Network Science, Yale University, New Haven, CT 06520 USA; 3 Department of Sociology, Yale University, New Haven, CT 06520 USA; 4 Department of Ecology and Evolutionary Biology, Yale University, New Haven, CT 06520 USA; 5 Department of Medicine, Yale University, New Haven, CT 06520 USA; 6 Department of Social Psychology, Tilburg University, Tilburg 5038AB, NL, The Netherlands; 7 Department of Biomedical Data Science, Geisel School of Medicine at Dartmouth, Lebanon, NH 03766, USA; 8 The Dartmouth Institute of Health Policy and Clinical Practice, Geisel School of Medicine at Dartmouth, Lebanon, NH 03766, USA; 9 Department of Psychology, Yale University, New Haven, CT 06511, USA; 10 Department of Economics, Yale University, New Haven, CT 06520 USA

## Abstract

Are cooperative decisions typically made more quickly or slowly than non-cooperative decisions? While this question has attracted considerable attention in recent years, most research has focused on one-shot interactions. Yet it is repeated interactions that characterize most important real-world social interactions. In repeated interactions, the cooperativeness of one’s interaction partners (the “social environment”) should affect the speed of cooperation. Specifically, we propose that *reciprocal* decisions (choices that mirror behavior observed in the social environment), rather than cooperative decisions per se, occur more quickly. We test this hypothesis by examining four independent decision time datasets with a total of 2,088 subjects making 55,968 decisions. We show that reciprocal decisions are consistently faster than non-reciprocal decisions: cooperation is faster than defection in cooperative environments, while defection is faster than cooperation in non-cooperative environments. These differences are further enhanced by subjects’ previous behavior – reciprocal decisions are faster when they are consistent with the subject’s previous choices. Finally, mediation analyses of a fifth dataset suggest that the speed of reciprocal decisions is explained, in part, by feelings of conflict – reciprocal decisions are less conflicted than non-reciprocal decisions, and less decision conflict appears to lead to shorter decision times.

Understanding the evolution of cooperation has been a major focus of research for decades^1^,^2^,^3^,^4^,^5^,^6^,^7^,^8^,^9^,^10^,^11^,^12^,^13^. Exploring the proximate cognitive mechanisms underlying this extraordinary cooperation helps to shed light on the evolutionary forces that gave rise to it^14^,^15^,^16^,^17^,^18^,^19^. In recent years, an emerging body of work has sought to illuminate the cognitive processes involved in cooperation by examining the speed at which humans make cooperative versus non-cooperative decisions^20^,^21^,^22^,^23^,^24^,^25^,^26^,^27^,^28^,^29^,^30^,^31^,^32^.

This work focused primarily, however, on one-shot games, asking if cooperative decisions are faster (or slower) than defection decisions. These studies have produced inconsistent results: although many find that cooperation is faster than defection^21^,^22^,^24^,^27^,^28^,^29^, others report the opposite pattern^20^,^23^,^26^. (Importantly, here we are referring to work examining *correlations* between decision speed and cooperation, rather than experimental *manipulations* of decision speed (or cognitive processing more generally) where the results are much more consistent: a recent meta-analysis of 51 manipulation studies with over 17,000 total participants shows that experimentally inducing intuitive decision-making has a clear positive effect on cooperation in 1-shot games^33^).

Despite prior work’s focus on one-shot games, life outside the laboratory is typified by repeated interactions over time, where there is a self-interested motivation to cooperate^6^,^8^,^9^,^34^. Thus, repeated interactions involve a conflict between the short-term gains from choosing defection and the long-term gains achieved through mutual cooperation^6^,^35^. Given the centrality of repeated interactions to social life, extending research on decision time correlations to repeated games may help to reconcile prior contradictory findings from one-shot games and further clarify the relationship between decision time and cooperation.

In the more ecologically valid context of repeated interactions, we propose that *reciprocity*, rather than cooperation or defection per se, occurs quickly. In repeated interactions, people are strongly influenced by the previous behavior of their interaction partners^36^,^37^,^38^,^39^. The norm of reciprocity is universal in human societies^40^ and it is an adaptive strategy in repeated interaction^9^,^41^. Critically, the hypothesis that reciprocity occurs quickly suggests that the social environment shapes the speed of cooperation. Hence, when people interact in a cooperative environment, their cooperation should be faster than defection. However, the opposite pattern should emerge when people interact in a non-cooperative environment – their defection should be faster than cooperation. The present study tests these predictions.

Furthermore, we shed light on precisely what the cognitive implications of decision time correlations are. Most prior work takes a *dual process* perspective, assuming that faster decisions are related to the use of automatic, intuitive process, whereas slower decisions are driven by deliberative, rational processes^42^,^43^,^44^,^45^. However, recent work^30^,^46^ has made the controversial argument that cooperative decision times are instead largely driven by *decision conflict*^47^,^48^,^49^. Under this interpretation, fast decisions occur when people strongly prefer one response, and decisions are slow when people find competing responses equally appealing. In the present work, we take advantage of the reciprocity perspective to provide additional evidence for the decision conflict theory of decision times.

## Materials and Methods

### Data Summary

To explore the role of social environment in shaping the relationship between decision times and reciprocity, we examine data from four independent studies in which subjects play repeated Prisoner’s Dilemma games (PD, Studies 1 and 3) or repeated Public Goods Games (PGG, Studies 2 and 4)^38^,^50^,^51^,^52^ (Table 1). These data represent all of the repeated game experiments previously conducted by our group in which decision times were recorded. In all four studies, subjects make a series of choices about whether to pay a cost in order to benefit one or more interaction partners. After each choice, subjects are informed about the choices of all their interaction partners. This means that after the first round of each game, subjects are aware of the social environment in which their interactions are occurring. In total, we analyze the data of four studies, 108 different sessions, 2,088 human subjects, and 55,968 cooperation decisions (nested in this order). Studies 1 through 3 and Study 5 were approved by the Harvard University Committee on the Use of Human Subjects, and Study 4 was approved by the Yale University Human Subjects Committee. All methods were carried out in accordance with the relevant guidelines.

### Inclusion criteria

The inclusion criteria for datasets in our analysis of repeated games are 1) the game structure is PD or PGG; 2) repeated interactions are observed (since decision time reflecting others’ previous moves is not examined in one-shot games); and 3) the defined decision time is adequately recorded (please see the definition below). Among studies fitting the first and the second condition, we excluded several potential sources of data^53^,^54^,^55^,^56^, because they did not meet the 3^rd^ condition. We thus obtained data of four independent studies implemented from 2007 to 2013 (Studies 1 to 4)^38^,^50^,^51^,^52^, which were briefly summarized in Table 1.

### Study 1

Dreber *et al*.^50^ recruited 104 Boston-area university students in the US, and investigated the effect of adding a costly punishment option into the typical two options (C or D) in the repeated PD on cooperation. The experiments took place at Harvard Business School Computer Lab for Experimental Research (HBS CLER). The recruited individuals joined one of a total of four sessions between April and May 2007, in which they were randomly assigned to a treatment session (a costly punishment option was added, i.e., C, D, or Punish, N = 54) or a control session (that option was not added; C or D only, N = 50). Since the costly punishment option was not the research focus of the present study, we used the data from the two control sessions. The subjects repeatedly interacted with a same individual in a PD up to 95 rounds via computer. Since interaction partners were shuffled several times during a single session, there were intermediate rounds without the cooperation history of interaction partners newly connected, which we omitted from the analysis. The contribution to the opponent was dichotomous: C or D. In the two control sessions, two different payoff matrices were applied (benefit-cost ratio [b/c] = 2 or b/c = 3). In total, we obtained 2,770 decision-making events in the conventional repeated PD with decision time.

### Study 2

Rand *et al*.^51^ recruited 192 subjects among Boston-area university students in the US, and investigated the role of an additional stage of reward and punishment after the stage of a typical PGG with repeated interactions. The experiments also took place at HBS CLER. The recruited individuals joined one of a total of 8 sessions between February and March 2009, in which the rules governing the additional stage was manipulated (stage 1 for the PGG, and stage 2 for punishment and reward to interaction partners): no additional stage, an additional stage of punishment, that of reward, or that of reward/punishment. They repeatedly interacted with the same individuals in a group of four subjects in a PGG up to 50 rounds via computer. Here the effective b/c was 2. Since the contribution to opponents was a continuous variable (contribute 0–20 monetary units), we created a dichotomous variable of C (contribution is 10 or more) or D (contribution is less than 10). Using another threshold for classifying cooperation v.s. defection (C for 20 and D for less than 20) does not substantially change the results (Table S8). In total, we obtained 9,600 decision-making events in the conventional repeated PD with decision time.

### Study 3

Fudenberg *et al*.^38^ recruited 384 Boston-area university students in the US, and investigated the evolution of cooperation when intended cooperative decision-making was implemented with noise added to the typical repeated PD. The experiments took place at Harvard University Decision Science Laboratory (DSL). The recruited individuals joined one of a total of 18 sessions between September 2009 and October 2010, in which the b/c ratio (four options: 1.5, 2.0, 2.5, or 4) and the error probability (three options) were manipulated. Subjects repeatedly interacted with a same individual in a PD up to 139 rounds via computer. Since interaction partners were shuffled several times during a single session, there were intermediate rounds without the cooperation history of interaction partners newly connected, which we omitted from the analysis. The contribution to the opponent was dichotomous: C or D. Due to the nature of the study, the actual decisions were not necessarily identical to the intended decisions. Since focal individuals could refer to the actual decision of the opponent at the last round, and decided on their intended decisions, we used the information of actual decisions for the type of social environment, and the intended decisions for the focal individuals’ decision-makings. In total, we obtained 30,038 decision-making events in the conventional repeated PD with decision time.

### Study 4

Nishi *et al*.^52^ recruited 1,462 subjects through Amazon Mechanical Turk (Mturk)^57^ from all over the world, and investigated the effect of endowment inequality and the information availability of network neighbors’ score (i.e., wealth) on the dynamics of cooperation and other outcomes. The recruited subjects joined one of a total of 80 online sessions between October and December 2013 and repeatedly interacted with connecting neighbors in a PGG up to 10 rounds via computer. The contribution to the public good (investment toward all the connecting neighbors) was dichotomous: “cooperate (C)” with all of them or “defect (D)” against all of a subject’s connections. The benefit-cost ratio (b/c) was 2. In total, we obtained 13,560 decision-making events in the PGG with decision time.

### Decision time

The main outcome variable in our analysis was decision time (the distribution is shown in Fig. S1). Decision time has commonly been used in basic and applied psychology^58^,^59^, and has been more commonly used in broader disciplines of social science in relation to neuroscience^22^,^60^,^61^,^62^,^63^. Decision time was previously defined as “the number of seconds between the moment that our server receives the request for a problem until the moment that an answer is returned to the server”^60^. Here, to fit the definition with our setting, we redefined decision time as the time between when a step in which each subject was asked to choose cooperate or defect appeared on the screen and when each subject clicked Cooperate or Defect on the screen, for example, in Study 4 (Fig. S3). Also, as indicated in prior literature^60^, the subjects were not informed that decision time was recorded in any of the four studies.

### Analytic procedure

Since the data regarding the decision-making events (Studies 1 to 4) were observed multiple times in a single subject, in a single session, and in a single study, we took into account the hierarchical data structure by using multilevel analysis with a random intercepts model (restricted maximum likelihood [REML])^64^, in the following statistical analyses for each study and for the combined data of the four studies (three levels for the study-specific analysis and four levels for the joint analysis; *P* values reported below are based on these models). For the outcome variable of the multilevel analysis, we log_10_-transformed the decision time (seconds), because the distribution of decision times was heavily right-skewed (the same transformation was used in prior work^22^,^63^).

We classified the decision-making of a focal individual in a given round into cooperative decisions (choosing to cooperate) and defection decisions (choosing to defect). Because baseline decision times varied considerably across experiments, we took the *percent* change in decision time of cooperation relative to defection (i.e. 100 × ([average decision time of cooperation] − [average decision time of defection])/[average decision time of defection]), rather than the absolute difference in decision times. We then examined the effect of social environment by comparing this difference in decision times for subjects who were in a cooperative versus non-cooperative social environment.

**Figure 1:**
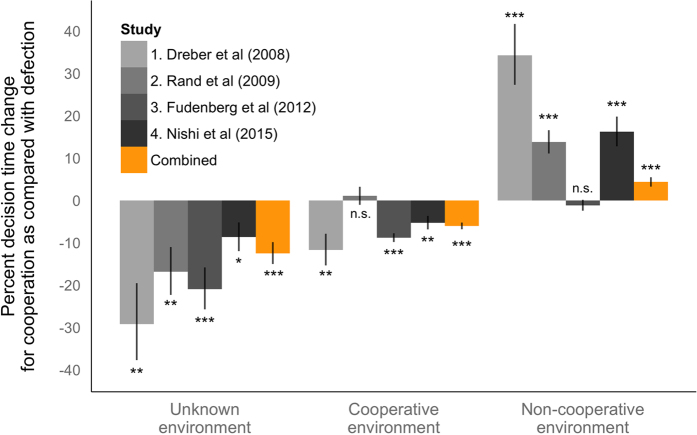
Cooperation is faster than defection in an unknown social environment and in a cooperative social environment, while defection is faster in a non-cooperative social environment across four studies of repeated economic games and in the combined data. The percent change in decision time for cooperation as compared with that for defection is calculated by regression analysis using random intercepts models that account for the hierarchical data structure (studies, sessions, individuals, and decisions). Left, the results in the 1^st^ round, in which subjects are in an unknown social environment and do not know if neighbors are cooperative or not, are shown. Middle, the results of cooperative social environments in later rounds (≥2) are shown. Right, the results of non-cooperative social environments in later rounds (≥2) are shown. A cooperative social environment is defined as cooperation rate of interaction partners at the last round of 0.5 or more, while a non-cooperative social environment is defined as that of less than 0.5. Error bars, point estimate ± standard error. n.s. for *P* ≥ 0.05, * for *P* < 0.05, ** for *P* < 0.01, and *** for *P* < 0.001.

For the data at the 1^st^ round (“unknown environment”), in each of the two categories (cooperation decisions or defection decisions), the relative difference of decision time was calculated (through exponentiation of the point estimates), and a *P* value for comparison between cooperation and defection decisions was calculated (N = 2,068 decisions) (Fig. 1, left). In the unknown environment, subjects make their choices without information regarding the previous behavior of their interaction partners (as is the case in previous work examining decision times in one-shot games).

**Figure 2:**
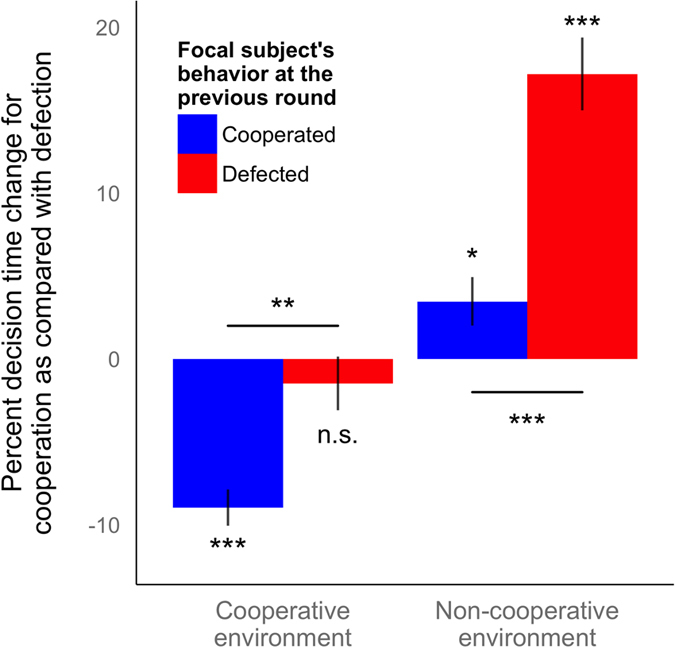
Speed of cooperation as compared with defection in cooperative environments is more clearly seen when subjects cooperate in the previous round, and speed of defection in non-cooperative environments is more clearly seen when subjects defect in the previous round. Using the combined data of the four studies, the percent change in decision time for cooperation as compared with that for defection at the present round is calculated by random intercepts model in the four categories: cooperators in the previous round facing cooperative social environments (left, blue), defectors in the previous round facing cooperative social environments (left, red), cooperators in the previous round facing non-cooperative social environments (right, blue), and defectors in the previous round facing non-cooperative social environments (right, red). Both the result of hypothesis testing for each bar (away from 0) and that for the comparison between two bars by an interaction term are shown. *P* values for the interaction term indicate the effect differs significantly between previous cooperators and defectors. Error bars, point estimates ± standard errors. n.s. for *P* ≥ 0.05, * for *P* < 0.05, ** for *P* < 0.01, and *** for *P* < 0.001.

For the data regarding the second round or later (N = 53,900 decisions), we classified the decision-making of interaction partner(s) at a previous round (i.e., type of social environment) into a cooperative environment (defined as cooperation rate of connecting neighbors at the previous move ≥ 0.5 or more) and a non-cooperative environment (the rate < 0.5) (sensitivity analyses using different thresholds did not substantially change the results) (Table S7). Note that, since the people to whom each subject connects is unique to each subject, the type of social environment (i.e. peers) varies at the subject level. We added a continuous variable of round number as a covariate for the multilevel analyses, since the decision time naturally decreases over the rounds (omitting round as a covariate does not substantially change the results). At each of the two-by-two categories (cooperation or defection decisions × cooperation or non-cooperative environments), the relative difference of decision time was calculated, and a *P* value for comparison between cooperation decisions and defection decisions was calculated (Fig. 1, middle and right). Also, in order to jointly investigate the difference of decision time between two decisions specific to an environment (cooperative or non-cooperative), we created an interaction term of the “decision” and “environment,” and calculated the *P* value of the term (Table S4). Moreover, we stratified the data after the second round by the cooperation decision at the previous round ([t − 1]^th^ round) and at the previous and first rounds (Figs 2 and S2).

We also performed another sensitivity analysis to examine the potential influence of variation in the b/c ratio (range: 1.5 to 4, but mostly 2), as b/c ratio has been shown to influence the speed of cooperation^46^. To show that our main results are not artifacts of variation in b/c, we demonstrate qualitatively similar results when restricting the data to only those conditions with a b/c ratio of 2 (i.e. excluding conditions from Studies 1 and 3 with b/c ≠ 2) (Table S10).

For the results in the figures, the coefficients calculated with the log_10_-transformed decision time were exponentiated back to report the percent change in decision time from defection decisions to cooperation decisions (we report only percent changes – i.e., ratio measures, which are robust to the “retransformation problem”^65^ affecting absolute values and differences, when assuming a homogenous variance).

### Additional analysis with Study 5

Finally, to shed light on the psychological processes underlying the speed of reciprocal decisions, we re-analyze reciprocity behavior in a one-shot asynchronous trust game. In Study 5, Evans *et al*.^30^ recruited 235 American subjects through Mturk, and investigated feelings of conflict and decision times for second movers in the trust game^66^. In the trust game, Player 1 (P1) can send 0, 10, 20, 30, or 40 cents to Player 2 (P2); any money sent is tripled by the experimenter; and then P2 decides how much of the tripled money (if any) to return to P1. The strategy selection method was used, meaning that P2 made a separate decision for each possible choice of P1. Before each decision, subjects were asked to rate how conflicted they felt, and P2’s responses to P1’s four non-zero decisions were presented in a random order.

Prior work shows that the more P1 sends, the stronger P2’s desire to return money^67^. Thus, in this one-shot game, the level of trust that P1 shows towards P2 forms P2’s social environment (more trust by P1 creates a more cooperative social environment for P2). This social environment is exogenously drawn from the P2 perspective. Inspired by recent theories of decision conflict as the driver of decision times in social dilemmas^25^,^30^,^46^, we hypothesize that in cooperative social environments, cooperative subjects will feel less conflicted, and thus decide more quickly, than non-cooperative subjects. In non-cooperative environments, conversely, we hypothesize that the opposite will be true. Moreover, we hypothesize that decision conflict will mediate the relationship between social environment and cooperation when predicting decision times. To test this hypothesis, we examine subjects’ responses to the question, “*How conflicted do you feel about your decision?*”, measured on the screen immediately prior to the final decision screen^30^.

Here, we estimated a multilevel model of moderated mediation where the interactive effects of social environment (initial trust) and P2 choice (amount returned to the first mover) on decision time were mediated by feelings of conflict (Fig. S4). Social environment and P2 choice were scaled to range from −0.5 to +0.5. Feelings of conflict were made on a scale from 1 to 10 and were z-transformed. The coefficients were estimated by generalized structural equation model estimation^68^.

### Data accessibility

The data reported in this paper are archived at Yale Institute for Network Science Data Archive and are available upon request.

## Results

Our results show that when subjects are deciding in the unknown environment, there is a negative relationship between decision time and cooperation across the four studies (Fig. 1, left). All four studies exhibit a significant relationship (*P* = 0.007, 0.006, < 0.001, and 0.014), and the combined data of the four also exhibit a significant relationship: cooperation decisions are 12.5% quicker than defection decisions (*P* < 0.001). Our analyses using the first-round data from studies with repeated interactions thus generally replicate the findings of prior studies investigating decision time in one-shot economic cooperation games^21^,^22^,^24^,^27^,^28^,^29^. All the analytic results are shown in Tables S1–S9.

For decisions beginning with the second round or later, our results show that social environment strongly moderates the relationship between decision time and cooperation: there is a significant interaction between social environment and decision (cooperate or defect) when predicting decision time in each of the four studies and in the combined data of the four studies (all interaction *P*s < 0.001) (Table S4). To understand this interaction, we test the relationship between cooperation and decision time within the cooperative and non-cooperative social environments separately.

When subjects are deciding in the context of a *cooperative environment*, there is a negative relationship between decision time and cooperation: cooperation decisions are significantly faster than defection decisions in three of the four studies (*P* = 0.003, 0.615, <0.001, and 0.001) (Fig. 1, middle). The combined data exhibit a significant relationship: cooperation decisions are 6.0% quicker than defection decisions overall (*P* < 0.001). The level of speed is similar to the results in the unknown environment (i.e., cooperation is 12.5% faster in an unknown environment at the 1^st^ round v.s. 6.0% faster in a cooperative environment at later rounds, adjusting for the round effect) (*P* = 0.957) (Table S9). This similarity suggests that, in an unknown environment, people are typically assuming that others will be cooperative.

Conversely, when subjects are deciding in the context of a *non-cooperative environment*, cooperation decisions are significantly *slower* than defection decisions in three of the four studies (*P* < 0.001, <0.001, 0.370, <0.001) (Fig. 1, right). The combined data also exhibit a significant relationship: cooperation decisions are 4.4% slower than defection decisions (*P* < 0.001). In sum, in both social environments, reciprocal decisions that mirrored the previous choices of interaction partners are faster than non-reciprocal decisions.

Furthermore, we investigate the interaction between the individual and their social environment. First, we ask how the subject’s own decision in the previous round influences decision times. In a cooperative environment, the subject’s previous behavior influences the speed of cooperation and defection decisions (interaction *P* = 0.003) (Fig. 2, left): previous cooperators are faster to choose cooperation than defection (9.0% difference, *P* < 0.001), whereas cooperation and defection are comparably fast for previous defectors (1.5% difference, *P* = 0.361). Previous behavior also influences the speed of cooperation and defection decisions in a non-cooperative environment (interaction *P* < 0.001) (Fig. 2, right): previous defectors are much faster to choose defection than cooperation (17.2% difference, *P* < 0.001). Previous cooperators are also faster to select defection than cooperation (3.5% difference, *P* = 0.016), though this effect was smaller than the effect for previous defectors.

We also replicate these results when using an individual’s cooperation decision in the very first round of the session, which is not influenced by the behavior of other players, and therefore can be viewed as a more pure proxy for subjects’ predisposition to cooperate (i.e. the extent to which they express the “cooperative phenotype”^69^). The role of first-round cooperation is minor after the stratification by the subject’s previous behavior as shown above. However, in a non-cooperative environment, cooperation decisions require more time among subjects who initially choose to cooperate but later choose to defect (“learned defectors”) compared to subjects who initially and previously choose to defect (“consistent defectors”) (interaction *P* = 0.010) (Fig. S2).

**Figure 3:**
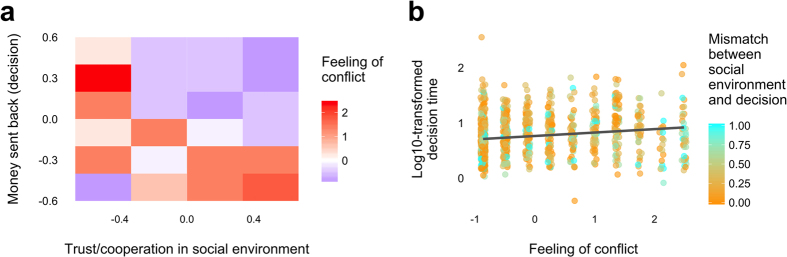
The mismatch between the social environment and decision relates to feelings of conflict (**a**), which can predict decision time (**b**) (Study 5). (**a**) Trust/cooperation in social environment (for Player 2) is proportional to the amount of money sent from Player 1 to Player 2. Both the measures of trust/cooperation in social environment (x-axis) and money sent back from Player 2 to Player 1 (y-axis) are standardized (range, −0.5 to 0.5). A higher value in both the measures represents a higher level of trust/cooperation to the opponent. Feeling of conflict (of Player 2) is the level of conflict when Player 2 decides the level of money sent back to Player 1 (y-axis) facing a certain level of trust in social environment (x-axis). A higher value in feeling of conflict represents a higher level of conflict. (**b**) Log_10_-transformed decision time (of Player 2) is the decision time when Player 2 decides the level of money sent back to Player 1. Mismatch between decision and environment is calculated by the absolute value of the difference between Level of trust in social environment and Level of money sent back (decision). The fitted line by simple linear regression is displayed to show the tendency.

Regarding the additional analysis of Study 5, we find that, when there is a mismatch between the P2’s social environment and P2’s decision (bottom-right and upper-left in Fig. 3a), P2 feels a higher level of conflict. Moreover, a higher level of conflict is associated with longer decision times (Fig. 3b). The structural equation model analyses support these findings: P2’s social environment (P1’s level of trust) and P2’s decision (amount P2 returns to P1) interact to determine feelings of conflict (*P* < 0.001) and decision times (*P* < 0.001) (Fig. S4). Importantly, feelings of conflict significantly *mediate* the interactive effects of social environment and P2’s decision on decision times (*P* = 0.001). As predicted, reciprocal choices (sending back large amounts of money after initial acts of trust) are less conflicted, and therefore, faster than non-reciprocal choices.

## Discussion

Here we have shown that in repeated interactions, reciprocal decisions occur more quickly: cooperation is faster than defection in cooperative social environments, while defection is faster than cooperation in non-cooperative environments. Therefore, it is not the case that cooperation is uniformly faster than defection, or vice versa. Interestingly, when subjects lack direct knowledge of their interaction partners (e.g., in an unknown environment), decision times are similar to those in the cooperative environment – cooperation is faster than defection. These findings are robustly observed in different repeated game types, conditions, time periods, and settings (both in-person and online). Similar results are also observed in the behavior of Player 2 in a one-shot Trust Game, where reciprocating is never payoff-maximizing (unlike in repeated games). This indicates that the relationship we observe is driven by an actual social preference for reciprocity (e.g., the willingness to incur a cost to reciprocate^70^,^71^,^72^), rather than just strategic reasoning in repeated games. Finally, we provide evidence that decision conflict drives our effect: reciprocal decisions are less conflicted than non-reciprocal decisions, and this lack of conflict explains a significant portion of the difference in decision times between reciprocal and non-reciprocal decisions.

Our results demonstrate the importance of considering social environment when examining decision time correlations, and may help to reconcile contradictory results from one-shot games. Expectations about interaction partners shape the relationship between decision time and cooperation. Hence, subjects’ beliefs about the likelihood of cooperation in one-shot games may produce positive, negative, or null correlations between decision time and cooperation. Consistent with this explanation, cooperation is typically faster than defection in one-shot game studies where most people cooperate (and therefore likely expected others to cooperate^22^,^24^,^27^), whereas defection is typically faster than cooperation in studies where defection is more common than cooperation^20^,^26^.

Our Study 5 adds support to a recent and unorthodox (within the cooperation literature) claim regarding the interpretation of decision times^30^,^46^: whereas many assume that faster decisions are more intuitive, we provide evidence that instead faster decisions are less *conflicted*. It seems natural that reciprocal decisions involve less decision conflict, as reciprocity is typically long-run payoff maximizing. Importantly, while intuition/deliberation and decision conflict have been shown to be dissociable processes^30^, the same logic that explains why reciprocity is low conflict *also* suggests that reciprocity should be intuitive^19^. And indeed, behavioral experiments which manipulate the use of intuition versus deliberation show that intuition favors both positive and negative reciprocity^73^,^74^,^75^,^76^.

Theories of spillover effects in laboratory experiments (e.g., the Social Heuristics Hypothesis^33^,^63^,^77^,^78^) emphasize that experiences from outside the lab influence subjects’ decisions and neurocognitive processes. The fact that, in the “unknown” environment, cooperation was faster than defection is consistent with the idea that daily experiences with norms and institutions initially led our American subjects to expect others to cooperate, and to be inclined towards cooperation themselves. However, once subjects engage in game play and learn about the behavior of their partners, they followed cues from the social environment. The initial expectation that others will cooperate comports well with, for example, evidence that American participants on Mturk tend to project a cooperative frame onto neutrally framed economic games^79^. It is also interesting to consider the connection between our results about baseline expectations and prior results suggesting that differences in baseline expectations about, and trust in, others influences participants’ intuitive default behaviors^22^,^80^,^81^.

Critically, our results are *not* consistent with the idea that simple imitation is what occurs quickly^82^. In particular, the interaction between social environment and the participant’s own move in the previous round (Fig. 2) highlights the role of reciprocal cooperation strategies, rather than simple imitation: imitation would lead to cooperation being faster than defection in a cooperative social environment (and defection being faster in a non-cooperative social environment) regardless of one’s previous move.

Our results also exclude the argument that faster responses are “error-prone”^83^, leading to a greater degree of mistakes in strategy implementation. On the contrary, we find that fast responses are *further* from random chance, and *more* in line with typically used (reciprocal) strategies: in cooperative social environments where most people cooperate, faster decisions are even more likely to be cooperative; and in non-cooperative environments, the opposite is true.

Although the experiments presented here involved humans making decisions in economic games played in the laboratory, our findings have implications beyond this setting. Firstly, there is substantial evidence that findings from laboratory games generalize to human behavior outside the lab^84^,^85^. Furthermore, decision speeds (often referred to as reaction times in the animal literature) are widely used in research on non-human animals, especially non-human primates, to make inferences about cognitive processes underlying decisions^86^,^87^,^88^, including specifically in the context of prosociality^89^. Our findings suggest that decision speed studies in non-human animals should not neglect the importance of social environment, and should consider the role of decision conflict (rather than different forms of cognitive processing) in determining decision speeds.

## Conclusion

Our results emphasize the centrality of reciprocity for human cooperation, and the importance of considering repeated games effects and associated variation in social environment when exploring the relationship between decision times and cooperation. Our results suggest that the speed of reciprocity is driven by (lack of) feelings of conflict (which is distinct from whether the actions are more intuitive versus deliberative^30^). Further specifying the neurocognitive mechanisms underlying quick reciprocal decisions is an important direction for future work; prior studies suggest the role of various brain areas for different types of reciprocal cooperation^36^,^90^,^91^,^92^. It would also be instructive to examine the role of social environment in the inferences people drawn based on others’ decision times^93^,^94^,^95^,^96^, and to explore whether the findings in the present study are observed in other primates^97^, in human children^98^,^99^, and in humans with a neurodevelopmental disorder such as autism^100^. When people are free to do as they choose, the thing they do most quickly is to reciprocate the behavior of others.

## Additional Information

**How to cite this article**: Nishi, A. *et al*. Social Environment Shapes the Speed of Cooperation. *Sci. Rep.* **6**, 29622; doi: 10.1038/srep29622 (2016).

## Supplementary Material

Supplementary Information

## Figures and Tables

**Table 1:** Characteristics of the four independent studies used.

Study No.	Author (year)	Study population	Games	Number of participants	Number of sessions	Number of decisions	Maximum rounds	Contribution	Research topic
1	Dreber *et al*.^50^	Boston-areauniversity students(HBS CLER)	PD	50	2	2,770	95	C/D**	Costly punishment
2	Rand *et al*.^51^	Boston-areauniversity students(HBS CLER)	PGG	192	8	9,600	50	0–20*	Reward and punishment
3	Fudenberg *et al*.^38^	Boston-areauniversity students(Harvard DSL)	PD	384	18	30,038	139	C/D	Noise in behaviors
4	Nishi *et al*.^52^	Amazon Mturkworkers	PGG	1,462	80	13,560	10	C/D	Endowment inequality

DSL, Decision Science Laboratory; HBS CLER, Harvard Business School Computer Lab for Experimental Research; Mturk, Mechanical Turk; PGG, Public goods game; PD, Prisoner’s dilemma game; C, Cooperation; D, Defection.

^*^10 or more is categorized as C, and less than 10 is categorized as D for the main analysis.

^**^The treatment group (n = 54) allowed subjects to have a third choice (punishment) in addition to C/D, and so we restricted our analysis to the control group (n = 50).

## References

[b1] Doebeli M. & Hauert C. Models of cooperation based on the Prisoner’s Dilemma and the Snowdrift game. Ecology letters 8, 748–766, 10.1111/j.1461-0248.2005.00773.x (2005).

[b2] Warneken F. & Tomasello M. Altruistic helping in human infants and young chimpanzees. Science 311, 1301–1303, 10.1126/science.1121448 (2006). 16513986

[b3] Clutton-Brock T. Cooperation between non-kin in animal societies. Nature 462, 51–57, 10.1038/nature08366 (2009). 19890322

[b4] Fehl K., van der Post D. J. & Semmann D. Co-evolution of behaviour and social network structure promotes human cooperation. Ecology Letters 14, 546–551, 10.1111/j.1461-0248.2011.01615.x (2011). 21463459

[b5] Archetti M. . Economic game theory for mutualism and cooperation. Ecology letters 14, 1300–1312, 10.1111/j.1461-0248.2011.01697.x (2011). 22011186

[b6] Rand D. G. & Nowak M. A. Human cooperation. Trends in cognitive sciences 17, 413–425, 10.1016/J.Tics.2013.06.003 (2013). 23856025

[b7] Powers S. T. & Lehmann L. The co-evolution of social institutions, demography, and large-scale human cooperation. Ecology letters 16, 1356–1364, 10.1111/ele.12178 (2013). 24015852

[b8] Trivers R. The evolution of reciprocal altruism. Quarterly Review of Biology 46, 35–57 (1971).

[b9] Axelrod R. & Hamilton W. D. The evolution of cooperation. Science 211, 1390–1396 (1981). 7466396 10.1126/science.7466396

[b10] Perc M. & Szolnoki A. Coevolutionary games–A mini review. Biosystems 99, 109–125 (2010). 19837129 10.1016/j.biosystems.2009.10.003

[b11] Nowak M. A. & Sigmund K. Evolution of indirect reciprocity. Nature 437, 1291–1298, 10.1038/nature04131 (2005). 16251955

[b12] Ostrom E. Governing the commons: The evolution of institutions for collective action. (Cambridge Univ Pr, 1990).

[b13] Janssen M. A., Holahan R., Lee A. & Ostrom E. Lab Experiments for the Study of Social-Ecological Systems. Science 328, 613–617 (2010). 20431012 10.1126/science.1183532

[b14] Tooby J. & Cosmides L. The Past Explains the Present - Emotional Adaptations and the Structure of Ancestral Environments. Ethology and Sociobiology 11, 375–424, 10.1016/0162-3095(90)90017-Z (1990).

[b15] Kiyonari T., Tanida S. & Yamagishi T. Social exchange and reciprocity: confusion or a heuristic? Evol Hum Behav 21, 411–427, 10.1016/S1090-5138(00)00055-6 (2000). 11146306

[b16] Bowles S. & Gintis H. Origins of human cooperation. Dahl Ws Env. 429–443 (2003).

[b17] Chudek M. & Henrich J. Culture-gene coevolution, norm-psychology and the emergence of human prosociality. Trends in cognitive sciences 15, 218–226, 10.1016/j.tics.2011.03.003 (2011). 21482176

[b18] Jordan J. J., Peysakhovich A. & Rand D. G. In The Moral Brain: Multidisciplinary Perspectives (eds Decety J. & Wheatley T. ) (MIT Press, 2015).

[b19] Bear A. & Rand D. G. Intuition, deliberation, and the evolution of cooperation. Proceedings of the National Academy of Sciences, 10.1073/pnas.1517780113 (2016). PMC4743833 26755603

[b20] Piovesan M. & Wengstrom E. Fast or fair? A study of response times. Econ Lett 105, 193–196, 10.1016/J.Econlet.2009.07.017 (2009).

[b21] Zaki J. & Mitchell J. P. Equitable decision making is associated with neural markers of intrinsic value. Proc Natl Acad Sci USA 108, 19761–19766, 10.1073/pnas.1112324108 (2011). 22106300 PMC3241792

[b22] Rand D. G., Greene J. D. & Nowak M. A. Spontaneous giving and calculated greed. Nature 489, 427–430, 10.1038/nature11467 (2012). 22996558

[b23] Fiedler S., Glockner A., Nicklisch A. & Dickert S. Social Value Orientation and information search in social dilemmas: An eye-tracking analysis. Organizational Behavior and Human Decision Processes 120, 272–284, 10.1016/j.obhdp.2012.07.002 (2013).

[b24] Lotito G., Migheli M. & Ortona G. Is cooperation instinctive? Evidence from the response times in a public goods game. Journal of Bioeconomics 15, 123–133 (2013).

[b25] Krajbich I., Oud B. & Fehr E. Benefits of Neuroeconomic Modeling: New Policy Interventions and Predictors of Preference. American Economic Review 104, 501–506, 10.1257/Aer.104.5.501 (2014).

[b26] Lohse J., Goeschl T. & Diederich J. Giving is a question of time: Response times and contributions to a real world public good. University of Heidelberg Department of Economics Discussion Paper Series (566) (2014).

[b27] Nielsen U. H., Tyran J. R. & Wengstrom E. Second thoughts on free riding. Econ Lett 122, 136–139, 10.1016/j.econlet.2013.11.021 (2014).

[b28] Cappelen A. W., Nielsen U. H., Tungodden B., Tyran J. R. & Wengström E. Fairness is intuitive. *Available at SSRN:* http://ssrncom/abstract=2430774 (2014).

[b29] Rand D. G., Fudenberg D. & Dreber A. It’s the thought that counts: The role of intentions in noisy repeated games. J Econ Behav Organ 116, 481–499 (2015).

[b30] Evans A. M., Dillon K. D. & Rand D. G. Fast but not intuitive, slow but not reflective: Decision conflict drives reaction times in social dilemmas. Journal of Experimental Psychology: General 144, 951–966 (2015). 26413891 10.1037/xge0000107

[b31] Rubinstein A. Instinctive and cognitive reasoning: a study of response times. The Economic Journal 117, 1243–1259 (2007).

[b32] Rubinstein A. Response time and decision making: An experimental study. Judgment and Decision Making 8, 540–551 (2013).

[b33] Rand D. G. Cooperation, fast and slow: Meta-analytic evidence for a theory of social heuristics and self-interested deliberation. Psychological Science, Pre-print available at SSRN: http://ssrn.com/abstract=2783800 (2016). 10.1177/0956797616654455 27422875

[b34] Dreber A., Fudenberg D. & Rand D. G. Who cooperates in repeated games: The role of altruism, inequity aversion, and demographics. J Econ Behav Organ 98, 41–55, 10.1016/j.jebo.2013.12.007 (2014).

[b35] Rachlin H. Altruism and selfishness. Behavioral and Brain Sciences 25, 239–250, 10.1017/S0140525x02000055 (2002). 12744145

[b36] Delgado M. R., Frank R. H. & Phelps E. A. Perceptions of moral character modulate the neural systems of reward during the trust game. Nature neuroscience 8, 1611–1618, 10.1038/nn1575 (2005). 16222226

[b37] Dal Bo, P. & Frechette G. R. The Evolution of Cooperation in Infinitely Repeated Games: Experimental Evidence. American Economic Review 101, 411–429, 10.1257/aer.101.1.411 (2011).

[b38] Fudenberg D., Rand D. G. & Dreber A. Slow to Anger and Fast to Forgive: Cooperation in an Uncertain World. American Economic Review 102, 720–749, 10.1257/aer.102.2.720 (2012).

[b39] Gallo E. & Yan C. The effects of reputational and social knowledge on cooperation. Proc Natl Acad Sci USA 112, 3647–3652, 10.1073/pnas.1415883112 (2015). 25775544 PMC4378402

[b40] Gouldner A. W. The Norm of Reciprocity - a Preliminary Statement. American Sociological Review 25, 161–178, 10.2307/2092623 (1960).

[b41] Rand D. G., Ohtsuki H. & Nowak M. A. Direct reciprocity with costly punishment: Generous tit-for-tat prevails. Journal of theoretical biology 256, 45–57, 10.1016/j.jtbi.2008.09.015 (2009). 18938180 PMC2614626

[b42] Sloman S. A. The empirical case for two systems of reasoning. Psychological Bulletin 119, 3 (1996).

[b43] Stanovich K. E. & West R. F. Individual differences in rational thought. J Exp Psychol Gen 127, 161–188, 10.1037/0096-3445.127.2.161 (1998).

[b44] Miller E. K. & Cohen J. D. An integrative theory of prefrontal cortex function. Annual review of neuroscience 24, 167–202, 10.1146/annurev.neuro.24.1.167 (2001). 11283309

[b45] Kahneman D. A perspective on judgment and choice: mapping bounded rationality. Am Psychol 58, 697–720, 10.1037/0003-066X.58.9.697 (2003). 14584987

[b46] Krajbich I., Bartling B., Hare T. & Fehr E. Rethinking fast and slow based on a critique of reaction-time reverse inference. Nature communications 6, 7455, 10.1038/Ncomms8455 (2015). PMC4500827 26135809

[b47] Diederich A. Decision making under conflict: Decision time as a measure of conflict strength. Psychonomic bulletin & review 10, 167–176, 10.3758/Bf03196481 (2003). 12747504

[b48] Einhorn H. J. & Hogarth R. M. Ambiguity and Uncertainty in Probabilistic Inference. Psychological review 92, 433–461, 10.1037//0033-295x.92.4.433 (1985).

[b49] Kleiman T. & Hassin R. R. Non-conscious goal conflicts. J Exp Soc Psychol 47, 521–532, 10.1016/j.jesp.2011.02.007 (2011).

[b50] Dreber A., Rand D. G., Fudenberg D. & Nowak M. A. Winners don’t punish. Nature 452, 348–351, 10.1038/nature06723 (2008). 18354481 PMC2292414

[b51] Rand D. G., Dreber A., Ellingsen T., Fudenberg D. & Nowak M. A. Positive Interactions Promote Public Cooperation. Science 325, 1272–1275 (2009). 19729661 10.1126/science.1177418 PMC2875121

[b52] Nishi A., Shirado H., Rand D. G. & Christakis N. A. Inequality and Visibility of Wealth in Experimental Social Networks. Nature 526, 426–429, 10.1038/nature15392 (2015). 26352469

[b53] Rand D. G., Arbesman S. & Christakis N. A. Dynamic Social Networks Promote Cooperation in Experiments with Humans. Proc Natl Acad Sci USA 108, 19193–19198, 10.1073/pnas.1108243108 (2011). 22084103 PMC3228461

[b54] Pfeiffer T., Tran L., Krumme C. & Rand D. G. The value of reputation. J R Soc Interface 9, 2791–2797, 10.1098/rsif.2012.0332 (2012). 22718993 PMC3479914

[b55] Shirado H., Fu F., Fowler J. H. & Christakis N. A. Quality Versus Quantity of Social Ties in Experimental Cooperative Networks. Nature communications 4, 2814, 10.1038/ncomms3814 (2013). PMC3868237 24226079

[b56] Rand D. G., Nowak M. A., Fowler J. H. & Christakis N. A. Static Network Structure Can Stabilize Human Cooperation. Proc Natl Acad Sci USA 111, 17093–17098, 10.1073/pnas.1400406111 (2014). 25404308 PMC4260616

[b57] Buhrmester M., Kwang T. & Gosling S. D. Amazon’s Mechanical Turk: A New Source of Inexpensive, Yet High-quality, Data? Perspect Psychol Sci 6, 3–5, 10.1177/1745691610393980 (2011). 26162106

[b58] Deary I. J., Liewald D. & Nissan J. A free, easy-to-use, computer-based simple and four-choice reaction time programme: The Deary-Liewald reaction time task. Behav Res Methods 43, 258–268, 10.3758/S13428-010-0024-1 (2011). 21287123

[b59] Kosinski R. A. A literature revew on reaction time (http://biae.clemson.edu/bpc/bp/lab/110/reaction.htm) (2013).

[b60] Rubinstein A. Instinctive and cognitive reasoning: A study of response times. Econ J 117, 1243–1259, 10.1111/J.1468-0297.2007.02081.X (2007).

[b61] Wansink B., Just D. R. & Payne C. R. Mindless Eating and Healthy Heuristics for the Irrational. American Economic Review 99, 165–169, 10.1257/Aer.99.2.165 (2009). 29505211

[b62] Zaki J. & Mitchell J. P. Intuitive Prosociality. Curr Dir Psychol Sci 22, 466–470, 10.1177/0963721413492764 (2013).

[b63] Rand D. G. . Social heuristics shape intuitive cooperation. Nature communications 5, 3677, 10.1038/Ncomms4677 (2014). 24751464

[b64] Graubard B. I. & Korn E. L. Modelling the sampling design in the analysis of health surveys. Statistical methods in medical research 5, 263–281 (1996). 8931196 10.1177/096228029600500304

[b65] Duan N. Smearing Estimate - a Nonparametric Retransformation Method. Journal of the American Statistical Association 78, 605–610, 10.2307/2288126 (1983).

[b66] Berg J., Dickhaut J. & Mccabe K. Trust, Reciprocity, and Social-History. Game Econ Behav 10, 122–142, 10.1006/game.1995.1027 (1995).

[b67] Pillutla M. M., Malhotra D. & Murnighan J. K. Attributions of trust and the calculus of reciprocity. J Exp Soc Psychol 39, 448–455, 10.1016/S0022-1031(03)00015-5 (2003).

[b68] Rabe-Hesketh S., Skrondal A. & Pickles A. Generalized multilevel structural equation modeling. Psychometrika 69, 167–190, 10.1007/Bf02295939 (2004).

[b69] Peysakhovich A., Nowak M. A. & Rand D. G. Humans display a ‘cooperative phenotype’ that is domain general and temporally stable. Nature communications 5, 4939, 10.1038/Ncomms5939 (2014). 25225950

[b70] Levine D. K. Modeling Altruism and Spitefulness in Experiments. Review of Economic Dynamics 1 (1998).

[b71] Dufwenberg M. & Kirchsteiger G. A theory of sequential reciprocity. Game Econ Behav 47, 268–298 (2004).

[b72] Falk A. & Fischbacher U. A theory of reciprocity. Game Econ Behav 54, 293–315 (2006).

[b73] Sutter M., Kocher M. & Straub S. Bargaining under time pressure in an experimental ultimatum game. Econ Lett 81, 341–347 (2003).

[b74] Smith P. & Silberberg A. Rational maximizing by humans (Homo sapiens) in an ultimatum game. Animal cognition 13, 671–677 (2010). 20130945 10.1007/s10071-010-0310-4

[b75] Grimm V. & Mengel F. Let me sleep on it: Delay reduces rejection rates in ultimatum games. Econ Lett 111, 113–115, 10.1016/j.econlet.2011.01.025 (2011).

[b76] Halali E., Bereby-Meyer Y. & Meiran N. Between Self-Interest and Reciprocity: The Social Bright Side of Self-Control Failure. Journal of experimental psychology: General 143, 745–754 (2014). 23895346 10.1037/a0033824

[b77] Peysakhovich A. & Rand D. G. Habits of virtue: Creating cultures of cooperation and defection in the laboratory. Management Science, 10.1287/mnsc.2015.2168 (2015).

[b78] Rand D. G., Brescoll V. L., Everett J. A. C., Capraro V. & Barcelo H. Social heuristics and social roles: Intuition favors altruism for women but not for men. Journal of Experimental Psychology: General 145, 389–396 (2016). 26913619 10.1037/xge0000154

[b79] Engel C. & Rand D. G. What does “clean” really mean? The implicit framing of decontextualized experiments. Econ Lett 122, 386–389, 10.1016/j.econlet.2013.12.020 (2014).

[b80] Rand D. G. & Kraft-Todd G. T. Reflection Does Not Undermine Self-Interested Prosociality. Front Behav Neurosci 8, 300 (2014). 25232309 10.3389/fnbeh.2014.00300 PMC4153292

[b81] Capraro V. & Cococcioni G. Social setting, intuition, and experience in laboratory experiments interact to shape cooperative decision-making. Proc Roy Soc B, 10.1098/rspb.2015.0237 (2015). PMC4528537 26156762

[b82] Heyes C. Automatic Imitation. Psychological bulletin 137, 463–483, 10.1037/a0022288 (2011). 21280938

[b83] Recalde M. P., Riedl A. & Vesterlund L. Error prone inference from response time: The case of intuitive generosity. *Available at SSRN*: http://ssrn.com/abstract=2507723 (2014).

[b84] Kraft-Todd G., Yoeli E., Bhanot S. & Rand D. Promoting cooperation in the field. Current Opinion in Behavioral Sciences 3, 96–101, 10.1016/j.cobeha.2015.02.006 (2015).

[b85] Englmaier F. & Gebhardt G. Social dilemmas in the laboratory and in the field. J Econ Behav Organ 128, 85–96, 10.1016/j.jebo.2016.03.006 (2016).

[b86] Roitman J. D. & Shadlen M. N. Response of neurons in the lateral intraparietal area during a combined visual discrimination reaction time task. The Journal of neuroscience 22, 9475–9489 (2002). 12417672 10.1523/JNEUROSCI.22-21-09475.2002 PMC6758024

[b87] Roesch M. R. & Olson C. R. Neuronal activity related to reward value and motivation in primate frontal cortex. Science 304, 307–310 (2004). 15073380 10.1126/science.1093223

[b88] Ratcliff R., Cherian A. & Segraves M. A comparison of macaque behavior and superior colliculus neuronal activity to predictions from models of two-choice decisions. Journal of neurophysiology 90, 1392–1407 (2003). 12761282 10.1152/jn.01049.2002

[b89] Chang S. W., Gariépy J.-F. & Platt M. L. Neuronal reference frames for social decisions in primate frontal cortex. Nature neuroscience 16, 243–250 (2013). 23263442 10.1038/nn.3287 PMC3557617

[b90] Rilling J. K. & Sanfey A. G. The Neuroscience of Social Decision-Making. Annual Review of Psychology Vol 62 62, 23–48, 10.1146/annurev.psych.121208.131647 (2011). 20822437

[b91] Sakaiya S. . Neural correlate of human reciprocity in social interactions. Front Neurosci-Switz 7, 239, 10.3389/fnins.2013.00239 (2013). PMC3865425 24381534

[b92] Watanabe T. . Two distinct neural mechanisms underlying indirect reciprocity. Proc Natl Acad Sci USA 111, 3990–3995, 10.1073/pnas.1318570111 (2014). 24591599 PMC3964069

[b93] Evans A. M. & Van de Calseyde P. P. F. M. The effects of observed decision time on expectations of extremity and cooperation. J Exp Soc Psychol (in press).

[b94] Jordan J. J., Hoffman M., Nowak M. A. & Rand D. G. Uncalculating Cooperation Is Used to Signal Trustworthiness. *Available at SSRN*: http://ssrn.com/abstract=2725550 (2016). 10.1073/pnas.1601280113 PMC4978259 27439873

[b95] Critcher C. R., Inbar Y. & Pizarro D. A. How quick decisions illuminate moral character. Social Psychological and Personality Science 4, 308–315 (2013).

[b96] Pizarro D., Uhlmann E. & Salovey P. Asymmetry in Judgments of Moral Blame and Praise: The Role of Perceived Metadesires. Psychological Science 14, 267–272, 10.1111/1467-9280.03433 (2003). 12741752

[b97] Fogassi L. . Parietal lobe: From action organization to intention understanding. Science 308, 662–667, 10.1126/science.1106138 (2005). 15860620

[b98] Falck-Ytter T., Gredeback G. & von Hofsten C. Infants predict other people’s action goals. Nature neuroscience 9, 878–879, 10.1038/nn1729 (2006). 16783366

[b99] Blake P. R., Rand D. G., Tingley D. & Warneken F. The shadow of the future promotes cooperation in a repeated prisoner’s dilemma for children. Scientific reports 5, 14559, 10.1038/srep14559 (2015). 26417661 PMC4586758

[b100] Dapretto M. . Understanding emotions in others: mirror neuron dysfunction in children with autism spectrum disorders. Nature neuroscience 9, 28–30, 10.1038/nn1611 (2006). 16327784 PMC3713227

