# Supplementary material for: Social Environment Shapes the Speed of Cooperation
Source: Sci Rep. 2016 Jul 20;6:29622. doi: 10.1038/srep29622 (PMC4951649; doi:10.1038/srep29622)
Supplement: Supplementary Information [file srep29622-s1.pdf]

Supplementary Information for
"Social Environment Shapes the Speed of Cooperation"

Akihiro Nishi, Nicholas A. Christakis, Anthony M. Evans, and A. James O'Malley, David G. Rand*

*To whom correspondence should be addressed. E-mail: david.rand@yale.edu (DGR).

A list of SI Appendix

Figs. S1 – S4 2
Tables S1 – S10 6

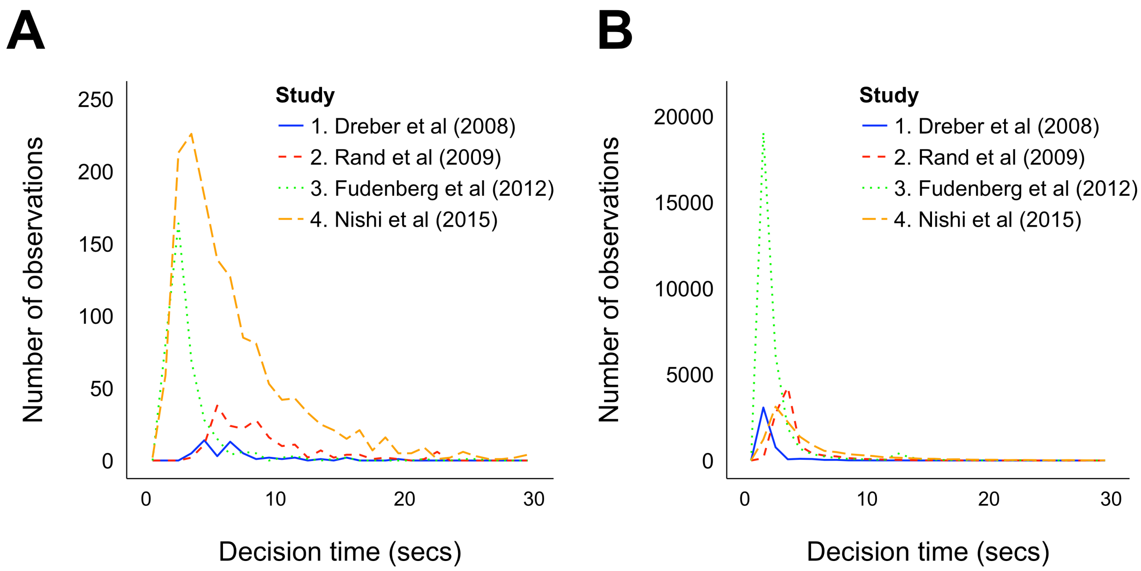

Fig. S1. Distributions of decision time in the four studies (frequency polygon plots). (A) 1st round. **(B)** 2nd- rounds.

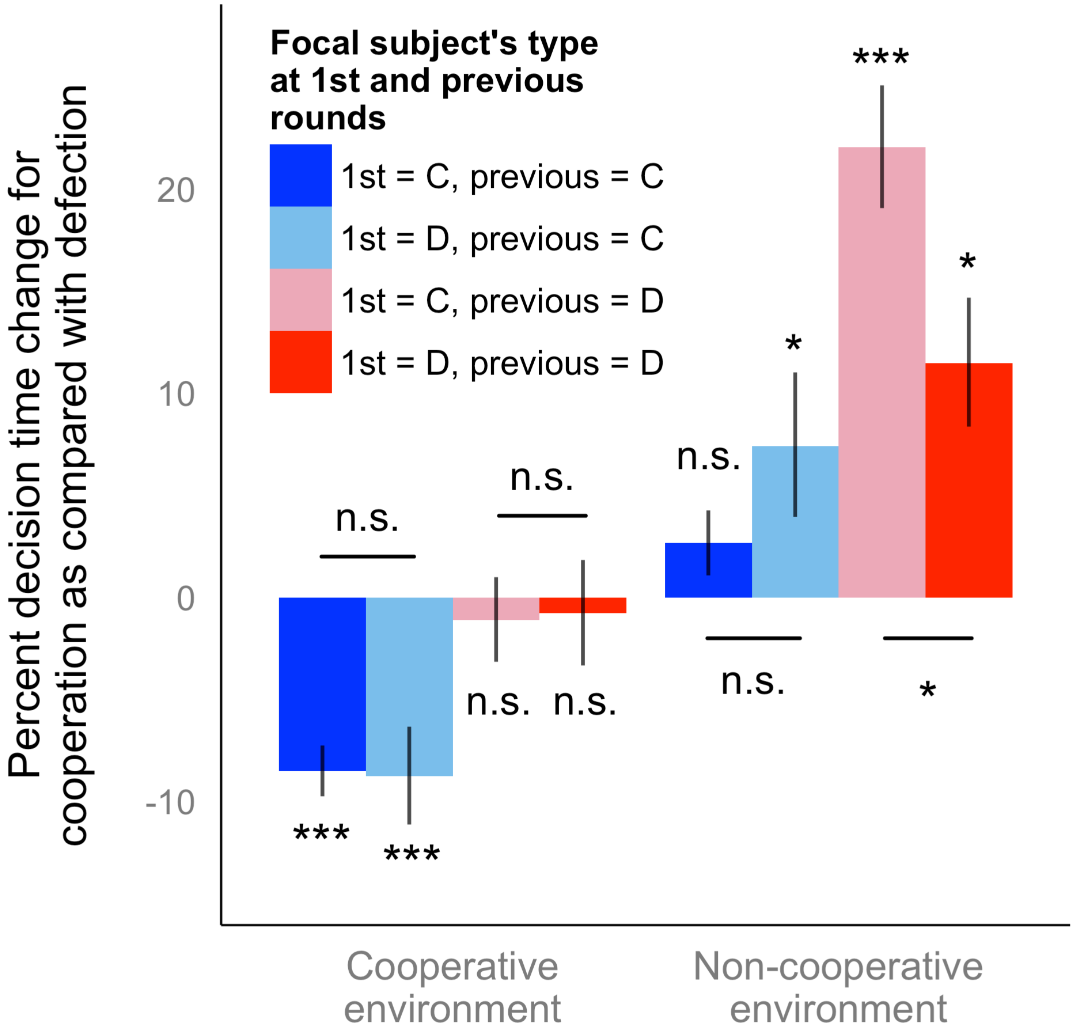

Fig. S2. In addition to Fig. 2 (stratification by the previous-round behaviors), statistical analysis was performed stratified by the 1st-round behaviors. C represents cooperation decisions, and D represents defection decisions. Both the result of hypothesis testing for each bar (away from 0) and that for the comparison between two bars by an interaction term are shown. Error bars, point estimates ± standard errors. n.s. for $P \geq 0.05$, * for $P < 0.05$, ** for $P < 0.01$, and *** for $P < 0.001$.

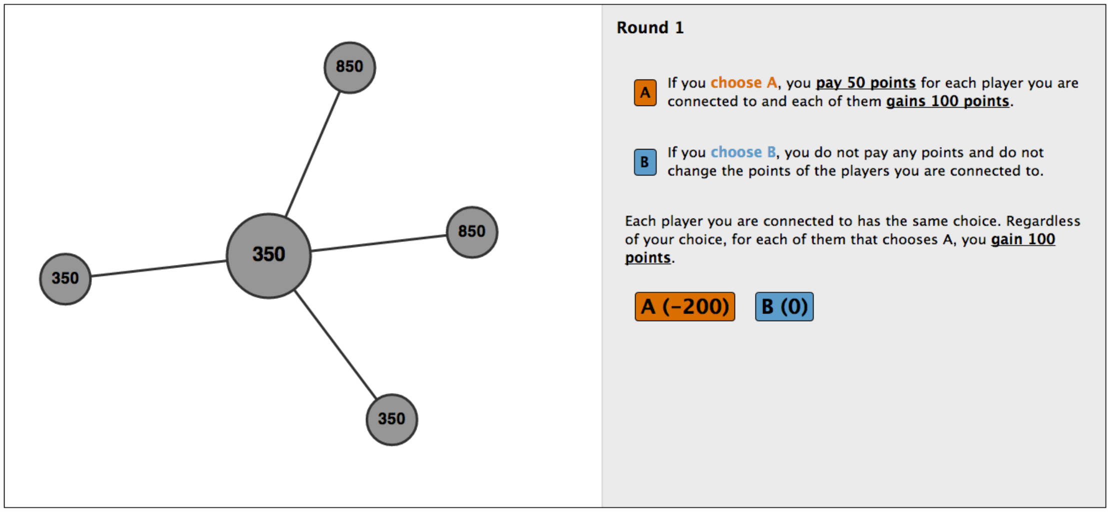

Fig. S3. Illustrative screenshot on when the decision time is measured (from Nishi et al, 2015 [Study 4]). In the screenshot, the focal individual having a score of 350 is asked to choose "A (-200)" (cooperate, "C") or "B (0)" (defect, ""D). Values in the circles represent the cumulative payoff at the 1st round of the focal individual and connecting. Decision time represents how long each individual stays at this screen. The one for the visible condition is shown (the scores of connecting neighbors are available), which was not shown in the invisible condition.

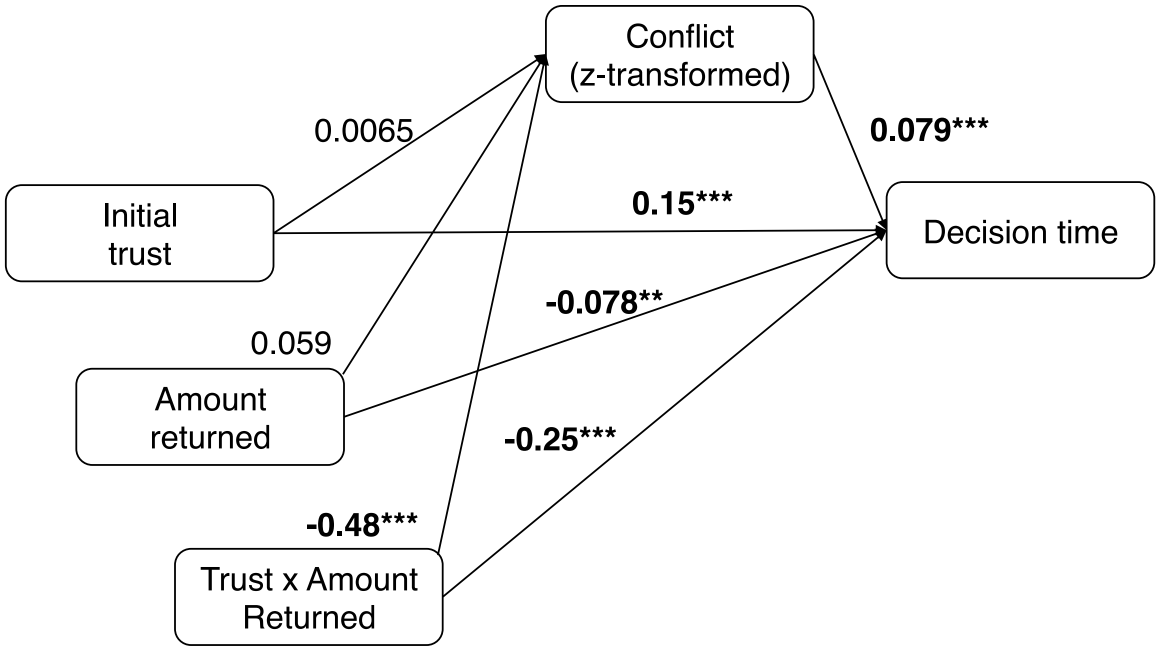

Fig. S4. Structural equation modeling shows the association of reciprocity (trust x amount returned) with decision time is partially mediated by level of conflict. Initial trust is the level of money sent from Player 1 (P1) to Player 2 (P2), which represents the type of the social environment of P1. Amount returned is the level of money sent back from P2 to P1, which represent the decision making of P2. The level of conflict of P2 is z-transformed, and decision time of P2 is $\log_{10}$-transformed. No sign for $P \geq 0.05$, ** for $P < 0.01$, and *** for $P < 0.001$.

	Study 1	Study 2	Study 3	Study 4	Combined
**Fixed effects**					
Cooperation	-0.150**	-0.0802**	-0.102***	-0.0393*	-0.0579***
	(0.0556)	(0.0295)	(0.0272)	(0.0161)	(0.0128)
Constant	0.866***	0.945***	0.429***	0.764***	0.725***
	(0.0564)	(0.0286)	(0.0322)	(0.0149)	(0.116)
**Random effects**					
Study-level variance					0.0522
					(0.0431)
Game-level variance	0.00208	0.00849	0.00997	0.00383	0.00486
	(0.00482)	(0.00314)	(0.00440)	(0.00133)	(0.00116)
Residual variance	0.0322	0.0251	0.0575	0.0783	0.0689
	(0.00663)	(0.00296)	(0.00426)	(0.00300)	(0.00221)
N	50	192	384	1442	2068

Table S1. Statistical analysis at the 1st round (the original results for Fig. 1, left). Standard errors in parentheses. For fixed effects, * $P < 0.05$, ** $P < 0.01$, and *** $P < 0.001$. Random intercepts model was used.

	Study 1	Study 2	Study 3	Study 4	Combined
**Fixed effects**					
Cooperation	-0.0540**	0.00459	-0.0400***	-0.0235**	-0.0270***
	(0.0184)	(0.00912)	(0.00488)	(0.00733)	(0.00366)
Round	-0.00233***	-0.000723***	0.000184***	-0.0119***	0.0000133
	(0.000340)	(0.000141)	(0.0000524)	(0.00101)	(0.0000498)
Constant	0.251***	0.513***	0.182***	0.712***	0.378**
	(0.0564)	(0.0130)	(0.0240)	(0.0104)	(0.124)
**Random effects**					
Study-level variance					0.0608
					(0.0502)
Game-level variance	0.00561	0.00296	0.00929	0.00158	0.00321
	(0.00831)	(0.000982)	(0.00343)	(0.000614)	(0.000610)
Subject-level variance	0.00256	0.00501	0.0137	0.0275	0.0220
	(0.00112)	(0.000736)	(0.00112)	(0.00149)	(0.000940)
Residual variance	0.0534	0.0266	0.0443	0.0575	0.0447
	(0.00260)	(0.000464)	(0.000492)	(0.000907)	(0.000356)
N	890	6748	16625	9418	33681

Table S2. Statistical analysis at a cooperation-rich environment (the 2nd- rounds) (the original results for Fig. 1, middle). Standard errors in parentheses. For fixed effects, * P < 0.05, ** P < 0.01, and *** P < 0.001. Random intercepts model was used.

	Study 1	Study 2	Study 3	Study 4	Combined
**Fixed effects**					
Cooperation	0.128***	0.0561***	-0.00505	0.0653***	0.0185***
	(0.0232)	(0.0105)	(0.00563)	(0.0131)	(0.00459)
Round	-0.00130***	-0.00153***	0.000625***	-0.00989***	0.000437***
	(0.000223)	(0.000273)	(0.0000673)	(0.00208)	(0.0000613)
Constant	0.167**	0.499***	0.144***	0.672***	0.334**
	(0.0609)	(0.0176)	(0.0239)	(0.0179)	(0.124)
**Random effects**					
Study-level variance					0.0603
					(0.0499)
Game-level variance	0.00696	0.00418	0.00907	0.000640	0.00432
	(0.0103)	(0.00193)	(0.00337)	(0.000752)	(0.000964)
Subject-level variance	0.00580	0.00656	0.0144	0.0252	0.0166
	(0.00144)	(0.00145)	(0.00122)	(0.00239)	(0.00105)
Residual variance	0.0446	0.0335	0.0611	0.0548	0.0558
	(0.00150)	(0.000936)	(0.000768)	(0.00170)	(0.000572)
N	1830	2660	13029	2700	20219

Table S3. Statistical analysis at a defection-rich environment (the 2nd- rounds) (the original results for Fig. 1, right). Standard errors in parentheses. For fixed effects, * $P < 0.05$, ** $P < 0.01$, and *** $P < 0.001$. Random intercepts model was used.

	Study 1	Study 2	Study 3	Study 4	Combined
**Fixed effects**					
Cooperation	0.118***	0.0592***	-0.0103*	0.0488***	0.0126**
	(0.0236)	(0.00908)	(0.00490)	(0.0125)	(0.00405)
Cooperative environment, A	0.0342*	0.0398***	0.0121**	0.0296***	0.0216***
	(0.0141)	(0.00985)	(0.00438)	(0.00861)	(0.00341)
Cooperation x A	-0.155***	-0.0541***	-0.0216***	-0.0729***	-0.0370***
	(0.0283)	(0.0124)	(0.00641)	(0.0139)	(0.00508)
Round	-0.00167***	-0.00103***	0.000377***	-0.0115***	0.000185***
	(0.000184)	(0.000124)	(0.0000414)	(0.000894)	(0.0000382)
Constant	0.183**	0.477***	0.156***	0.682***	0.345**
	(0.0587)	(0.0119)	(0.0239)	(0.0116)	(0.124)
**Random effects**					
Study-level variance					0.0606
					(0.0500)
Game-level variance	0.00655	0.00307	0.00945	0.00146	0.00302
	(0.00956)	(0.000961)	(0.00346)	(0.000544)	(0.000559)
Subject-level variance	0.00418	0.00543	0.0132	0.0264	0.0208
	(0.00102)	(0.000709)	(0.00103)	(0.00131)	(0.000838)
Residual variance	0.0480	0.0287	0.0523	0.0571	0.0494
	(0.00131)	(0.000422)	(0.000432)	(0.000782)	(0.000307)
N	2720	9408	29654	12118	53900

Table S4. Statistical analysis for interactions (the 2nd- rounds). “Cooperation x A” is the variable of interest. Standard errors in parentheses. For fixed effects, * $P < 0.05$, ** $P < 0.01$, and *** $P < 0.001$. Random intercepts model was used.

	Cooperative environment			Non-cooperative environment		
	All	C at previous round	D at previous round	All	C at previous round	D at previous round
**Fixed effects**						
Cooperation at last round, A	0.0120*			-0.0304***		
	(0.00604)			(0.00506)		
Cooperation at present round, B	-0.0135*	-0.0408***	-0.00650	0.0546***	0.0148*	0.0688***
	(0.00574)	(0.00521)	(0.00713)	(0.00764)	(0.00613)	(0.00813)
A x B	-0.0234**			-0.0360***		
	(0.00776)			(0.00988)		
Round	0.0000156	0.0000841	-0.0000488	0.000431***	-0.000220*	0.000821***
	(0.0000498)	(0.0000565)	(0.000110)	(0.0000613)	(0.0000961)	(0.0000802)
Constant	0.375**	0.386**	0.383**	0.342**	0.354**	0.317**
	(0.124)	(0.128)	(0.122)	(0.123)	(0.126)	(0.121)
**Random effects**						
Study-level variance	0.0607	0.0653	0.0582	0.0592	0.0626	0.0574
	(0.0501)	(0.0538)	(0.0483)	(0.0491)	(0.0517)	(0.0477)
Game-level variance	0.00319	0.00301	0.00450	0.00430	0.00376	0.00489
	(0.000606)	(0.000634)	(0.00105)	(0.000965)	(0.00107)	(0.00111)
Subject-level variance	0.0219	0.0212	0.0216	0.0163	0.0141	0.0161
	(0.000938)	(0.00102)	(0.00149)	(0.00104)	(0.00133)	(0.00115)
Residual variance	0.0447	0.0392	0.0590	0.0556	0.0477	0.0585
	(0.000356)	(0.000360)	(0.00101)	(0.000570)	(0.000895)	(0.000734)
N	33676	25651	8025	20216	6561	13655

Table S5. Stratified analysis by the previous-round behaviors (the original results for Fig. 2). Standard errors in parentheses. For fixed effects, * $P < 0.05$, ** $P < 0.01$, and *** $P < 0.001$. Random intercepts model was used.

	Cooperative environment						Non-cooperative environment					
	C at previous round			D at previous round			C at previous round			D at previous round		
	All	C at first round	D at first round	All	C at first round	D at first round	All	C at first round	D at first round	All	C at first round	D at first round
**Fixed effects**												
Cooperation at first round, A	-0.0201			-0.0566***			-0.0131			-0.0332**		
	(0.0136)			(0.0125)			(0.0138)			(0.0109)		
Cooperation at present round, B	-0.0424***	-0.0387***	-0.0398***	0.00239	-0.00481	0.00341	0.0314*	0.0114	0.0311*	0.0474***	0.0865***	0.0472***
	(0.0109)	(0.00591)	(0.0114)	(0.0108)	(0.00910)	(0.0113)	(0.0126)	(0.00674)	(0.0143)	(0.0117)	(0.0107)	(0.0123)
A x B	0.00347			-0.00804			-0.0209			0.0416*		
	(0.0124)			(0.0144)			(0.0144)			(0.0162)		
Round	0.0000832	0.0000862	0.0000603	-0.0000323	0.0000416	-0.0000950	-0.000228*	-0.000219*	-0.000271	0.000822***	0.000736***	0.000898***
	(0.0000565)	(0.0000600)	(0.000164)	(0.000110)	(0.000152)	(0.000156)	(0.0000962)	(0.000103)	(0.000239)	(0.0000802)	(0.000113)	(0.000113)
Constant	0.401**	0.385**	0.395**	0.414***	0.352**	0.431***	0.364**	0.351**	0.367**	0.336**	0.307*	0.338**
	(0.129)	(0.127)	(0.136)	(0.120)	(0.126)	(0.110)	(0.126)	(0.124)	(0.127)	(0.119)	(0.124)	(0.114)
**Random effects**												
Study-level variance	0.0655	0.0633	0.0723	0.0560	0.0619	0.0469	0.0628	0.0609	0.0624	0.0559	0.0602	0.0502
	(0.0540)	(0.0522)	(0.0603)	(0.0464)	(0.0515)	(0.0394)	(0.0519)	(0.0502)	(0.0527)	(0.0464)	(0.0500)	(0.0422)
Game-level variance	0.00296	0.00327	0.00333	0.00424	0.00467	0.00434	0.00361	0.00275	0.00712	0.00469	0.00444	0.00521
	(0.000628)	(0.000744)	(0.00143)	(0.000988)	(0.00145)	(0.00150)	(0.00105)	(0.00105)	(0.00293)	(0.00108)	(0.00130)	(0.00154)
Subject-level variance	0.0212	0.0215	0.0186	0.0204	0.0150	0.0258	0.0141	0.0146	0.0130	0.0160	0.0142	0.0186
	(0.00102)	(0.00115)	(0.00233)	(0.00144)	(0.00182)	(0.00249)	(0.00133)	(0.00162)	(0.00267)	(0.00115)	(0.00149)	(0.00196)
Residual variance	0.0392	0.0386	0.0426	0.0590	0.0545	0.0630	0.0477	0.0435	0.0611	0.0584	0.0528	0.0636
	(0.000360)	(0.000385)	(0.00101)	(0.00101)	(0.00141)	(0.00145)	(0.000895)	(0.000936)	(0.00235)	(0.000734)	(0.000963)	(0.00110)
N	25641	21658	3983	8019	3714	4305	6561	5003	1558	13655	6581	7074

Table S6. Stratified analysis by the 1st-round and previous-round behaviors (the original results for Fig. S2). Standard errors in parentheses. For fixed effects, * *P* < 0.05, ** *P* < 0.01, and *** *P* < 0.001. Random intercepts model was used.

	Threshold = 0.4	Threshold = 0.5	Threshold = 0.6	Threshold = 0.7	Threshold = 0.8	Threshold = 0.9
**Fixed effects**						
Cooperation	0.0121**	0.0126**	0.0179***	0.0183***	0.0163***	0.0143***
	(0.00418)	(0.00405)	(0.00383)	(0.00360)	(0.00342)	(0.00328)
Cooperative environment, A	0.0210***	0.0216***	0.0241***	0.0232***	0.0221***	0.0193***
	(0.00336)	(0.00341)	(0.00348)	(0.00358)	(0.00369)	(0.00382)
Cooperation x A	-0.0351***	-0.0370***	-0.0475***	-0.0502***	-0.0489***	-0.0470***
	(0.00513)	(0.00508)	(0.00500)	(0.00491)	(0.00486)	(0.00490)
Round	0.000184***	0.000185***	0.000189***	0.000190***	0.000192***	0.000193***
	(0.0000382)	(0.0000382)	(0.0000382)	(0.0000382)	(0.0000382)	(0.0000382)
Constant	0.344**	0.345**	0.345**	0.346**	0.347**	0.347**
	(0.124)	(0.124)	(0.124)	(0.124)	(0.124)	(0.123)
**Random effects**						
Study-level variance	0.0604	0.0606	0.0609	0.0609	0.0606	0.0600
	(0.0499)	(0.0500)	(0.0502)	(0.0502)	(0.0500)	(0.0495)
Game-level variance	0.00303	0.00302	0.00301	0.00300	0.00300	0.00300
	(0.000560)	(0.000559)	(0.000557)	(0.000555)	(0.000555)	(0.000556)
Subject-level variance	0.0208	0.0208	0.0207	0.0206	0.0207	0.0207
	(0.000839)	(0.000838)	(0.000837)	(0.000835)	(0.000836)	(0.000837)
Residual variance	0.0494	0.0494	0.0494	0.0493	0.0493	0.0493
	(0.000307)	(0.000307)	(0.000307)	(0.000307)	(0.000307)	(0.000307)
N	53900	53900	53900	53900	53900	53900

Table S7. Sensitivity analysis 1: Threshold of neighbors' cooperation rates (0.4 – 0.9; 0.5 is used for the main analysis). "Cooperation x A" is the variable of interest. Standard errors in parentheses. The result of the threshold = 0.5 is the same as the one at Table S4, All (RI). For fixed effects, * $P < 0.05$, ** $P < 0.01$, and *** $P < 0.001$. Random intercepts model was used.

	C as ≥10	C as =20
**Fixed effects**		
Cooperation	0.0592***	0.00148
	(0.00908)	(0.0137)
Cooperative environment, A	0.0398***	0.0431***
	(0.00985)	(0.00775)
Cooperation x A	-0.0541***	-0.0512***
	(0.0124)	(0.0148)
Round	-0.00103***	-0.000902***
	(0.000124)	(0.000125)
Constant	0.477***	0.507***
	(0.0119)	(0.0114)
**Random effects**		
Game-level variance	0.00307	0.00292
	(0.000961)	(0.000916)
Subject-level variance	0.00543	0.00518
	(0.000709)	(0.000679)
Residual variance	0.0287	0.0287
	(0.000422)	(0.000422)
N	9408	9408

Table S8. Sensitivity analysis 2: Threshold of continuous variable of cooperation at Rand et al (Study 2). “Cooperation x A” is the variable of interest. Among the continuous donation to the public: 0 – 20 in the public goods game, “C as ≥10” represents that the threshold for the cooperators is a half contribution (≥10), while “C as =20” represents that the threshold is a full contribution (=20). Standard errors in parentheses. For fixed effects, * $P < 0.05$, ** $P < 0.01$, and *** $P < 0.001$. Random intercepts model was used.

	Unknown vs Cooperative	Unknown vs Non-cooperative
**Fixed effects**		
Cooperation	-0.0312***	0.0619***
	(0.00486)	(0.00704)
Indicator variable for Round = 1, A	0.161***	0.234***
	(0.00944)	(0.0107)
Cooperation x A	0.000611	-0.0857***
	(0.0114)	(0.0139)
Round (continuous), B	-0.000107	0.000596***
	(0.0000858)	(0.0000672)
Cooperation x B	0.0000366	-0.00104***
	(0.0000938)	(0.000124)
Constant	0.394***	0.330**
	(0.119)	(0.118)
**Random effects**		
Study-level variance	0.0557	0.0550
	(0.0460)	(0.0456)
Game-level variance	0.00340	0.00440
	(0.000635)	(0.000845)
Subject-level variance	0.0213	0.0161
	(0.000891)	(0.000956)
Residual variance	0.0460	0.0569
	(0.000355)	(0.000561)
N	35749	22287

Table S9. Additional analysis for the Unknown environment (1st round) v.s. the Cooperative environment (2nd- rounds) and for the Unknown environment (1st round) v.s. the Non-cooperative environment (2nd- rounds). “Cooperation x A” is the variable of interest. Standard errors in parentheses. For fixed effects, * *P* < 0.05, ** *P* < 0.01, and *** *P* < 0.001. Random intercepts model was used.

	1st round	2nd round or later		
		Cooperative environment	Non-cooperative	All
**Original results (all b/c ratios [1.5 - 4])**	ref: Table S1	ref: Table S2	ref: Table S3	ref: Table S4
Effect of cooperation	-0.0579***	-0.0270***	0.0185***	
	(0.0128)	(0.0037)	(0.0044)	
Cooperation x Cooperative environment				-0.0370***
				(0.0051)
N	2068	33681	20219	53900
**Additional results (b/c ratio = 2)**				
Effect of cooperation	-0.0458**	-0.0146**	0.0417***	
	(0.0144)	(0.0050)	(0.0067)	
Cooperation x Cooperative environment				-0.0524***
				(0.0076)
N	1714	18776	8670	27446

Table S10. Original results (all b/c ratios [1.5 - 4]) v.s. additional results (b/c ratio = 2). The original results are obtained from Tables S1 to S4. The main effects in the original and additional analyses are shown. For fixed effects, * $P < 0.05$, ** $P < 0.01$, and *** $P < 0.001$. Random intercepts model was used.
